# Supplementary material for: Ecology and life history of Meta bourneti (Araneae: Tetragnathidae) from Monte Albo (Sardinia, Italy)
Source: PeerJ. 2018 Nov 29;6:e6049. doi: 10.7717/peerj.6049 (PMC6275118; doi:10.7717/peerj.6049)
Supplement: Table S3 — Besides the inclusion of sector’s depth as a further independent variable, these analyses resemble those included in the manuscript in both used variables and methodology. [file peerj-06-6049-s003.docx]

|  | **Independent variables included into the model** | | | | | | | | | | | | **df** | **AICc** | **∆-AICc** | **Weight** |
| --- | --- | --- | --- | --- | --- | --- | --- | --- | --- | --- | --- | --- | --- | --- | --- | --- |
|  | **Height** | **Width** | **Het** | **Season** | **Cave** | **Temp** | **Hum** | **Lux** | **Temp˟S** | **Hum˟S** | **Lux˟S** | **Depth** |  |  |  |  |
| GLM |  |  | | | | | | | | | | | | | | |
| *Meta* spiders | | | | | | | | | | | | | | | | |
|  | **X** |  |  | **X** | **X** |  | **X** |  |  |  |  |  | **11** | **149.3** | **0** | **0.339** |
|  | X |  | X | X | X |  | X |  |  |  |  |  | 12 | 150.9 | 1.53 | 0.158 |
|  | X | X |  | X | X |  | X |  |  |  |  |  | 12 | 151 | 1.66 | 0.148 |
|  | X |  |  | X | X |  | X | X |  |  |  |  | 12 | 151.3 | 2.02 | 0.124 |
|  | X |  |  | X | X |  | X |  |  |  |  | X | 12 | 151.5 | 2.12 | 0.117 |
| Adults |  |  | | | | | | | | | | | | | | |
|  | **X** |  |  | **X** | **X** |  |  | **X** |  |  |  |  | **11** | **77.3** | **0** | **0.233** |
|  |  |  | X | X | X |  |  | X |  |  |  |  | 11 | 77.5 | 0.22 | 0.209 |
|  | X |  | X | X | X |  |  | X |  |  |  |  | 12 | 77.7 | 0.45 | 0.186 |
|  |  |  |  | X | X |  |  | X |  |  |  |  | 10 | 78.2 | 0.95 | 0.145 |
|  | X | X |  | X | X |  |  | X |  |  |  |  | 12 | 78.5 | 1.15 | 0.131 |
| Juveniles |  |  | | | | | | | | | | | | | | |
|  | **X** |  |  | **X** | **X** |  | **X** |  |  |  |  |  | **11** | **102.5** | **0** | **0.308** |
|  | X |  |  | X | X | X | X |  |  |  |  |  | 12 | 103.1 | 0.56 | 0.233 |
|  | X |  |  | X | X |  | X | X |  |  |  |  | 12 | 104.4 | 1.93 | 0.117 |
|  | X | X |  | X | X |  | X |  |  |  |  |  | 12 | 104.5 | 1.96 | 0.116 |
|  | X |  | X | X | X |  | X |  |  |  |  |  | 12 | 104.5 | 1.99 | 0.114 |
