# Supplementary material for: Ecology and life history of Meta bourneti (Araneae: Tetragnathidae) from Monte Albo (Sardinia, Italy)
Source: PeerJ. 2018 Nov 29;6:e6049. doi: 10.7717/peerj.6049 (PMC6275118; doi:10.7717/peerj.6049)
Supplement: Table S4 — For each group (A–C) are shown the significance of variables included in the relative best AICc model of the respective analysis. [file peerj-06-6049-s004.docx]

|  |  | **GLM** | | |
| --- | --- | --- | --- | --- |
|  | **Factor** | ***β*** | ***χ^2^*** | ***P*** |
| ***Meta bourneti*** |  |  |  |  |
|  | Season |  | 4.99 | 0.173 |
|  | Cave |  | 12.08 | **0.034** |
|  | Height | 0.27 | 17.51 | **<0.001** |
|  | Humidity | 11.23 | 9.64 | **0.002** |
| **Adults** |  |  |  |  |
|  | Season |  | 0.86 | 0.834 |
|  | Cave |  | 5.64 | 0.342 |
|  | Height | 0.24 | 3.75 | 0.053 |
|  | Illuminance | -3.03 | 10.06 | **0.001** |
| **Juveniles** |  |  |  |  |
|  | Season |  | 8.9 | **0.031** |
|  | Cave |  | 14.14 | **0.015** |
|  | Height | 0.28 | 13.73 | **<0.001** |
|  | Humidity | 13 | 8.19 | **0.004** |
